# Kava Components Down-Regulate Expression of AR and AR Splice Variants and Reduce Growth in Patient-Derived Prostate Cancer Xenografts in Mice

**DOI:** 10.1371/journal.pone.0031213

**Published:** 2012-02-09

**Authors:** Xuesen Li, Zhongbo Liu, Xia Xu, Christopher A. Blair, Zheng Sun, Jun Xie, Michael B. Lilly, Xiaolin Zi

**Affiliations:** 1 Department of Urology, University of California Irvine, Orange, California, United States of America; 2 Department of Pharmaceutical Sciences, University of California Irvine, Orange, California, United States of America; 3 Department of Pharmacology, University of California Irvine, Orange, California, United States of America; 4 Department of Medicine, University of California Irvine, Orange, California, United States of America; 5 Chao Family Comprehensive Cancer Center, University of California Irvine, Orange, California, United States of America; Baylor College of Medicine, United States of America

## Abstract

Men living in Fiji and drinking kava have low incidence of prostate cancer (PCa). However, the PCa incidence among Fijian men who had migrated to Australia, increased by 5.1-fold. We therefore examined the potential effects of kava root extracts and its active components (kavalactones and flavokawains) on PCa growth and androgen receptor (AR) expression. PCa cell lines (LNCaP, LAPC-4, 22Rv1, C4-2B, DU145 and PC-3) with different AR expression, and a transformed prostate myofibroblast cell line (WPMY-1), were treated with a commercial kava extract, kavalactones (kawain, 5′6′-dehydrokawain, yangonin, methysticin) and flavokawain B. Expression of AR and its target genes (*PSA* and *TMPRSS2*) was examined. Two novel patient-derived PCa xenograft models from high grade PCa specimens were established by implanting the specimens into nude mice and passing tumor pieces through subcutaneous injection in nude mice, and then treated with kava extract and flavokawain B to examine their effects on tumor growth, AR expression and serum PSA levels. The kava extract and flavokawain B effectively down-regulated the expression of both the full-length AR and AR splice variants. The kava extract and kavalactones accelerated AR protein degradation, while flavokawain B inhibited *AR* mRNA transcription via decreasing Sp1 expression and the binding of Sp1 to the AR promoter. The kava root extract and flavokawain B reduce tumor growth, AR expression in tumor tissues and levels of serum PSA in the patient-derived PCa xenograft models. These results suggest a potential usefulness of a safe kava product or its active components for prevention and treatment of advanced PCa by targeting AR.

## Introduction

Asian/Pacific men who consume a low fat and plant-based diet have the lowest rates of clinical PCa in the world [1], [2]. However, when Asian men migrate to the US, rates of clinical PCa increase [3]. These observations implicate both environmental factors and dietary habits (such as consumption of low-fat and plant-based diet) in PCa development. Therefore, one of strategies for prevention and treatment of PCa has focused on the use of natural and synthetic bioactive food components.

Kava (*Piper methysticum Forst*) is a perennial plant indigenous to the Pacific Islands. Kava root and rhizome are used to prepare a non-fermented and ceremonial beverage with relaxant effects in the Pacific Islands for thousands of years [4]. Unusually low incidences of several cancers, including lung and prostate cancer, are reported in the Pacific Island nations despite a high portion of smokers in these populations [2], [5]. In addition, Steiner [6] reported that the age-standardized cancer incidence for the three highest kava-drinking countries (Vanuatu, Fiji, and Western Samoa) was one fourth or one third that of non-kava-drinking countries, such as New Zealand and United States (Hawaii and Los Angeles), and non-kava-drinking Polynesians (Maoris). Uniquely, in these three kava-drinking countries more men drink kava and smoke than do women, yet there is a lower incidence of cancer for men than for women [6]. Moreover, the Cancer Council's Cancer in New South Wales (NSW) Migrants 1991–2001 report found [7] that the PCa incidence in Fijian men who migrated to and were resident in NSW, Australia, increased by 5.1 times compared to those living in Fiji. This report has prompted us to investigate the potential benefits of kava extracts and its active components for PCa prevention.

We report for the first time that a commercial kava extract and its active components (kavalactones and flavokawain B) down-regulate AR expression. Depletion of AR protein occurred through two different mechanisms: 1) enhanced AR protein degradation, and 2) reduced Specificity protein 1 (Sp1)-mediated AR transcription. Kava extract and flavokawain B treatment of mice with patient-derived xenografts reduced tumor growth and expression of AR and its target genes in tumor tissues, and lowered serum PSA levels.

## Methods

### Ethics statement

Use of mice and their care for this study was specifically approved by the University of California, Irvine Institutional Animal Care and Use Committee (IACUC; protocol number 2007–2741).

### Cell lines, compounds and reagents

The LNCaP, LAPC4, 22Rv1, PC3, DU145, and WPMY-1 cell lines were obtained from American Type Culture Collection (ATCC) (Manassas, VA), and C4-2B cell line was from Urocor Inc. (Oklahoma City, OK). These cells were cultured in RPMI 1640 medium with 10% fetal bovine serum (FBS). All cell lines used in this study were within 20 passages after receipt. The cell lines were tested and authenticated by ATCC or Urocor Inc. All cells lines were also tested for known species of mycoplasma contamination using a kit from LONZA Inc. (Walkersville, MD). Pure kawain, 5′, 6′-dehydrokawain, yangonin, methysticin, and flavokawain B (99%) were isolated from kava extracts by LKT Laboratories, Inc. (St. Paul, MN). Kava root extract at a concentration of 150 mg/ml kavalactones in 50% ethanol was obtained from Gaia Herbs (Brevard, NC). Antibodies against AR and tubulin were from Santa Cruz Biotechnology, Inc. (Santa Cruz, CA). PSA and Sp1 antibodies were purchased from Thermo Scientific (Fremont, CA). 3-(4, 5-dimethylthiazol-2-yl)-2, 5-diphenyltetrazolium bromide (MTT) was from Sigma. RNAzol B was purchased from Tel-Test (Friendswood, TX.). The Reverse Transcription System kit and was from Promega (Mandison, WI). A quantitative RT-PCR kit was from Bio-Rad (Hercules, CA).

### Measurement of kavalactones and flavokawains in the kava root extract

The kava extract was diluted 400 times by acetronitrile, and then filtered with 0.45 µm solvent resistant filters and stored at −80°C until further analysis. Chromatography was performed on an Acquity Ultra Performance Liquid Chromatography (UPLC) system (Waters Corp., Milford, MA, USA) with an auto-sampler at 8°C. Separation of compounds was carried out at 50°C using a Waters Acquity UPLC ®BEH C18 1.7 µm (2.1×50 mm) column with a gradient elution: (A) Water: Acetonitrile: acetic acid (97.8∶2∶0.2 v/v/v), and (B) Acetonitrile: acetic acid 99.8∶0.2 (v/v) as the mobile phase.

The elution program was as follows: 10% B (initial), 90% B (1.0 min), 90% B (2.0 min), 10%B (2.05 min), and 10% (3 min). The flow rate was 0.3 mL/min and the injection volume was 10 µL. The UPLC was coupled to Micromass Quattro Micro Liquid chromatography–mass spectrometry (LC/MS/MS) triple quadrupole mass spectrometer (Mass Range: 2∼2000 m/z) with electrospray ionization (ESI) interface in positive mode. The instrument was operated in a positive ion mode with an ESI voltage of 3.8 kV, and a desolvation gas flow of 700 L/h. Argon was used as the collision induced dissociation gas at a pressure of 7.1^e−3^ mbar with a selected reaction monitoring transition for flavokawain A of m/z 315>m/z 181, flavokawain B of 284>181, kawain of 231>115, 5′, 6′-dehydrokawain of 229>131, mythysticin of 275>159, Yangonin of 259>161.

### MTT assay

Cells were plated at a density of 2×10^4^ per well in 24-well culture plates for 24 hours, and then treated as indicated in the figures. After treatment for 72 hours, 1 mg/mL MTT was added to each well for 2 hours, and the absorbance was determined at 570 nm. Dose–response curves for growth inhibition were generated as a percentage of vehicle-treated controls.

### Western blot analysis

Clarified protein lysates (20–100 µg) were denatured and resolved by 8–16% SDS-PAGE. Proteins were transferred to nitrocellulose membranes, and probed with indicated antibodies and visualized by an enhanced chemiluminescence detection system.

### Quantitative RT-PCR

Real-time quantitative PCR amplification reactions for *AR*, prostate specific antigen (*PSA*) and Transmembrane protease, serine 2 (*TMPRSS2*) mRNA levels were carried out using MyiQ system (Bio-Rad) as described previously [8], [9]. The sequences of primers for *AR*, *PSA* and *TMPRSS2* are available upon request. Data were analyzed by using the comparative Ct method, where Ct is the cycle number at which fluorescence first exceeds the threshold. The Ct values from each sample were obtained by subtracting the values for beta-actin Ct from the target gene Ct value. The variation of *beta actin* Ct values is <0.5 among different samples. A one cycle difference of Ct value represents a 2-fold difference in the level of mRNA. Specificity of resulting PCR products was confirmed by melting curves and agarose gel.

### Transfection, promoter activity and luciferase Assay

The PSA-Luc and plARS-Luc plasmids are a kindly gift from Dr. Wang Longgui (New York University Cancer Institute). Human Sp1-HA tagged plasmid was kindly provided by Macus Tien Kuo (The University of Texas M. D. Anderson Cancer Center). C4-2B and LNCaP cells were co-transfected with PSA-Luc or plARS-Luc and Renilla luciferase plasmid pGL 4.71 (Promega) or with Sp1-HA plasmid by Lipofectamine 2000 (Invitrogen). After 48 hours, flavokawain B was added as indicated with triple replications. Then cells were harvested and luciferase activity was measured with the Dual-Glo Luiferase assay system (Promega). Renilla luminescence was used as an inner control for cell numbers and transfection efficiency. The relative ratio of luminescence from interested gene promoter to Renilla luminescence was shown in the figures as promoter activity. For Sp1 transfection, cells were harvest for an immunoblotting assay.

### Chromatin Immunoprecipitation (ChIP) [10]

LNCaP and C4-2B cells were cross-linked by formaldehyde. The protein/DNA complex was sheared to 500–1500 bp fragments by sonication. Equal amounts of protein (4 mg) were incubated with Sp1 antibody, or with a mouse anti-GFP antibody. Protein G sepharose beads (Sigma) was employed to pull down the complex, followed by washing with High Salt Wash Buffer, LiCl, and TE buffers). The DNA-protein immunoprecipitates were eluted from the beads and the DNA was extracted and purified. Expand High-Fidelity PCR System (Roche) was used to amplify the sequence from +248 to +487 nucleotides of the AR 5′-UTR region, containing two Sp1 binding sites. Primer sequences are available upon request.

### Treatment of patient-derived PCa tumor xenograft models

Prostatectomy tissues were obtained through an IRB-approved protocol at UCI Medical Center. Small fragments of histologically-proven tumor were implanted into 8-week-old male Severe combined immunodeficiency (SCID) mice using a subrenal xeongraft technique [11]. Tumors designated as GM0308 and RC0309 at 3 and 13 murine passages respectively, were re-implanted into subcutaneous pockets of SCID mice. One week later, when the tumors grew to about 100 mm^3^, mice were randomized into treatment groups. For the kava extract treatment, mice were fed a standard diet supplemented with either vehicle control or 6 g/kg kava extract in the diet. For the flavokawain B treatment, mice were injected intraperitoneally each day with vehicle control (10% DMSO, 20% ethanol and 70% Cremophor) or 200 mg flavokawain B/kilogram mouse body weight. Treatment was continued from day 0 to day 17 for the kava extract and to day 27 for flavokawain B. Tumor growth was monitored by both serum PSA and caliper measurement of tumor size weekly. Body weight and food intakes were recorded during the experiments. At the end of the experiments, tumor tissues were weighed and cut in half for mRNA isolation and immunohistochemistry staining, respectively. The tumor volume was calculated by the formula: 0.5236×L_1_×(L_2_)^2^, where L_1_ is the long axis and L_2_ is the short axis of the tumor. Serum PSA concentration was determined by PSA Enzyme-linked immunosorbent assay kit (Bio-Quant, San Diego, CA) following the kit's instruction.

### Immunohistochemistry

Antigen was retrieved using 0.05 M Glycine-HCL buffer, pH 3.5, containing 0.01% (w/v) EDTA, at 95°C for 20 min and stained with anti-human AR (1∶100). Staining was visualized with diaminobenzadine using the Cell and Tissue Staining kit (R&D Systems).

### Statistical analysis

Comparisons of quantitative RT-PCR and promoter assays between treatment and control experiments were conducted using Student's *t* test. For tumor growth experiments, repeated-measures ANOVA was used to examine the differences in tumor sizes among treatments, time points, and treatment-time interactions. Additional post-tests were done to examine the differences in tumor sizes between control, the kava extract, and flavokawain B treatment at each time point by using the conservative Bonferroni method. All statistical tests were two sided.

## Results

### The kava extract and its active components (kavalactones and flavokawain B) differentially inhibit the growth of AR expressing cells

**Figure 1:**
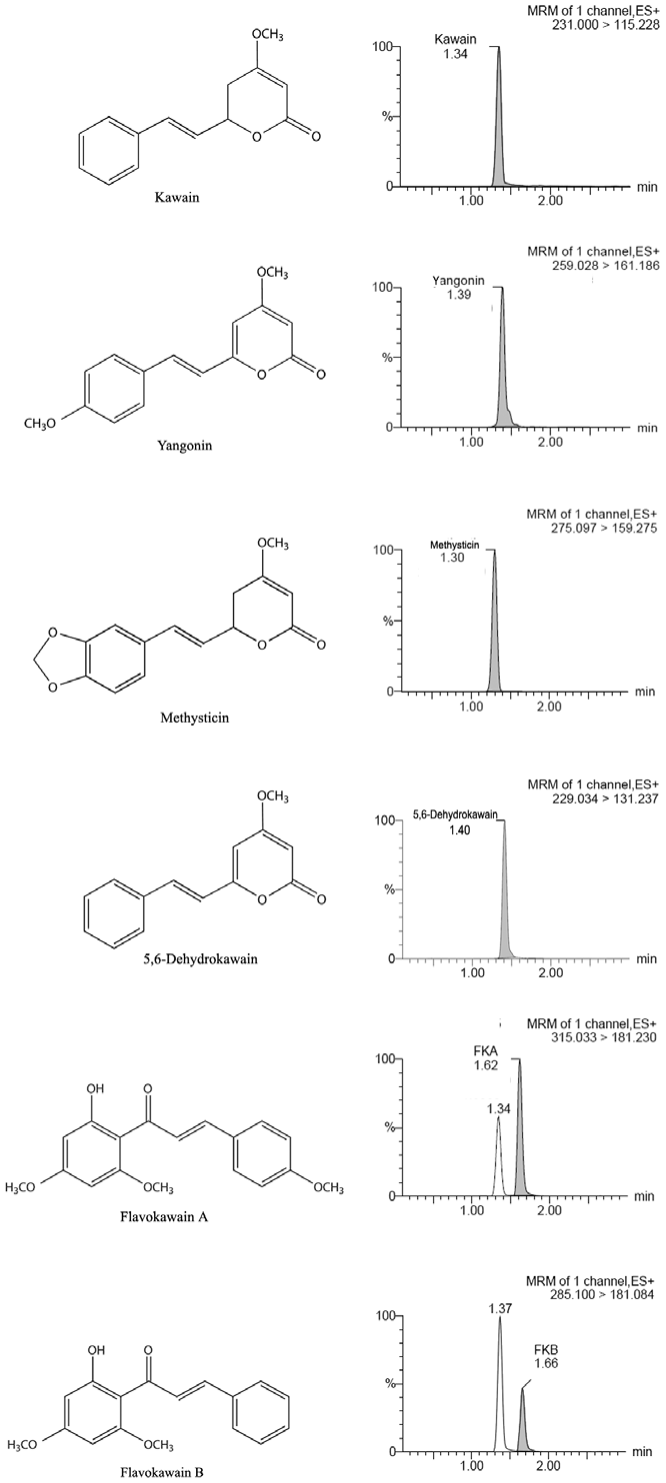
UPLC analysis of kavalactones and flavokawain B in the kava root extract.

**Figure 2:**
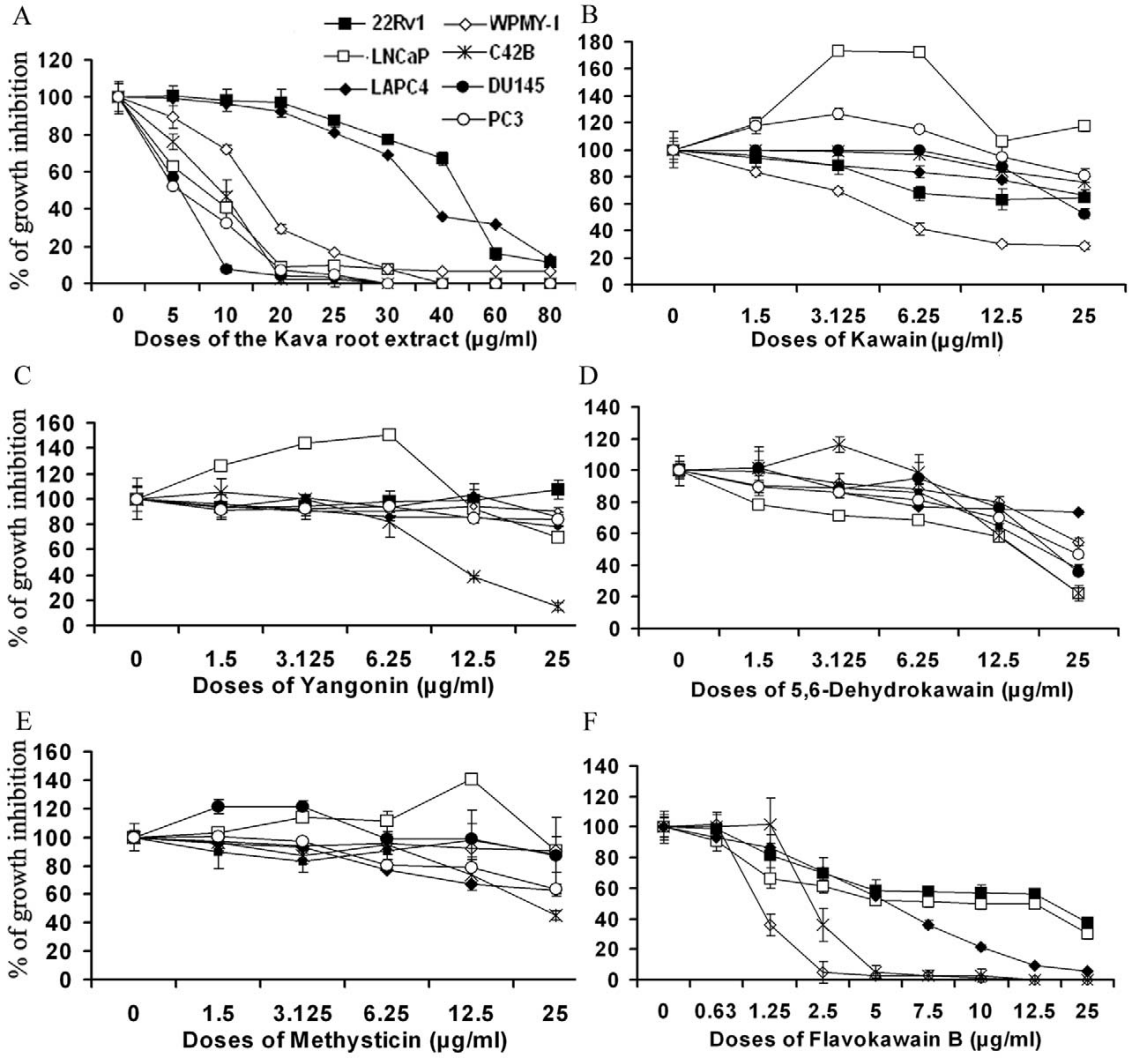
The effect of the kava root extract and its active components on the growth of PCa cell lines (C4-2B, LNCaP, 22Rv1, LAPC-4, DU145 and PC3) and a prostate myofibroblast WPMY-1. Cells in 24-well culture plates were treated with 0.1% DMSO, the kava root extract, kawain, 5′, 6′-dehydrokawain, yangonin, methysticin, or flavokawain B (FKB) at the indicated doses. After 72 hours of treatment, cell densities were measured by MTT assay. Each point is the mean of values from four independent plates; bars, SD. Each sample was counted in duplicate. IC_50 s_ were estimated by dose-response curves.

Kavalactones and chalcones (i.e. flavokawains) are two important classes of bioactive compounds identified from kava extracts. Using UPLC, we measured the contents of kavalactones and flavokawains. The commercial kava extract dissolved in ethanol and used here contained 2.7% kawain, 1.75% 5, 6-dehydrokawain, 3.08% Yangonin, 1.4% methysticin, 0.33% flavokawain B, and 0.21% flavokawain A (Figure 1). To examine the potential of the kava extract components to inhibit PCa growth, PCa cell lines and a transformed prostate myofibroblast line were treated with the kava extract, kavalactones and flavokawain B. The cell lines vary in their expression of AR proteins, as well as their androgen dependence. Figure 2 and Table 1 show that the kava root extract similarly inhibited androgen sensitive and insensitive PCa cell lines. The kava root extract was more potent than kavalactones but less potent than flavokawain B in inhibiting the growth. Among the kavalactones, 5, 6-dehydrokawain is the most potent agent in inhibiting the growth of PCa cell lines (Figure 2 and Table 1). Both the kava root extract and 5, 6-dehydrokawain equally in inhibiting the growth of LNCaP and its derivative C4-2B, while flavokawain B was about 4.5-times more effective in reducing the growth of C4-2B cells than the growth of LNCaP cells. Given that the kava extract has much less flavokawain B and more 5, 6-dehydrokawain, this result suggest that 5, 6-dehydrokawain or its combination with other active components may play a dominant role for the inhibitory effect of the kava extract on the growth of PCa cell lines.

**Table 1:** The IC_50 s_ of the kava root extract and its active components and AR status in prostate cell lines.

Cell lines	LNCaP	LAPC4	22Rv1	C4-2B	WPMY-1	DU145	PC3
Estimated IC_50_ (µg/mL)							
Kava root extract	6.5	35.7	46	7	15	5.4	5.3
Kawain	≫25	≫25	≫25	≫25	5.21	25	≫25
Yangonin	≫25	≫25	>25	7.8	≫25	≫25	≫25
5′6′Dehydrokawain	15.2	≫25	18.75	15.3	25	20.6	22.9
Methysticin	≫25	≫25	≫25	23	≫25	≫25	≫25
Flavokawain B	10	5.6	16.6	2.2	2.25	1.5	2.1
AR status*	MT	WT	SP	MT	WT	ND	ND
Androgen dependency#	AS	AD/AS	AS/AI	AI	AS	AI	AI

*AR status: MT: mutant; SP: splicing variant; WT: wild-type; ND: not detectable.

# Androgen dependency: AD: androgen is required for growth; AS: cells may respond to androgen, but do not require it for growth; AI: cells do not respond to androgen.

### Kava root extract and kavalactones decrease the expression of PSA and TMPRSS2 through acceleration of AR protein degradation

**Figure 3:**
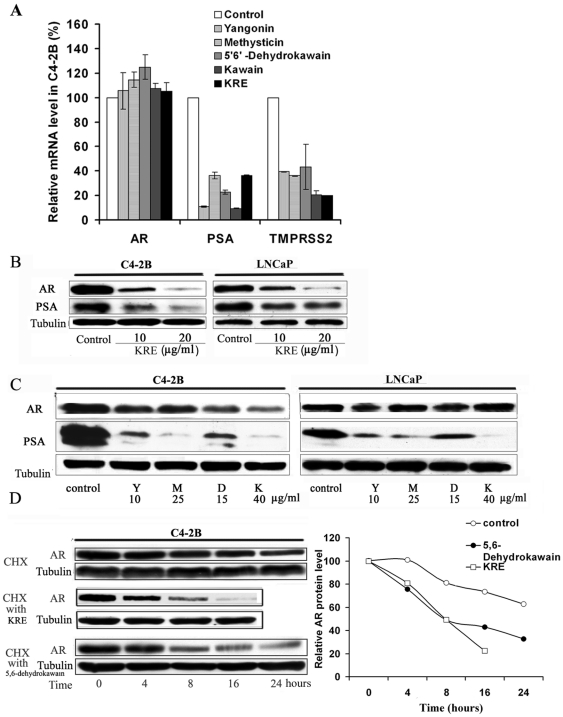
The kava root extract and kavalactones down-regulate expression of AR and AR target genes (*PSA* and *TMPRSS2*) via accelerating the degradation of AR protein. (**A**). C4-2B cells were treated with 0.1%DMSO, kava root extract, dehydrokawain, kawain, yangonin and methysticin for 8 hours. mRNA levels of AR and AR target genes (*PSA* and *TMPRSS2*) were determined by real-time RT-PCR. Bars are means ± SD of three independent quantitative measures. Kava root extract and kavalactones significantly decrease mRNA expression of AR target genes (Student *t* test, P<0.01) but not that of AR (P>0.05). (**B**) and (**C**). The protein expression of AR and PSA in LNCaP and C4-2B cells after indicated treatments at a concentration of their IC_50 s_ for 16 hours was analyzed by Western blot. α-Tubulin was detected as a loading control. A representative blot was shown from three independent experiments. Y = yangonin; M = methysticin; D = 5′6;-dehydrokawain; K = kawain, KRE = kava root extract. (**D**). C4-2B cells were pretreated with 10 µg/ml cycloheximide for 2 hours and then supplemented with kava root extract or 5′6;-dehydrokawain at a concentration of their IC_50 s_ for different periods of time. After treatments, AR protein levels were analyzed by Western blotting and semi-quantified by densitometry measurement. A representative blot was shown from three independent experiments.

Figure 3A shows that the kava extract and kavalactones (i.e. kawain, 5′6′-dehydrokawain, yangonin, and methysticin) markedly down-regulate the mRNA expression of AR target genes (*PSA* and *TMRSS2*) but not mRNA expression of AR in C4-2B cells. Figure 3B and C shows that both the kava extract and individual kavalactones decrease AR and PSA protein expression as well. The growth inhibitory effect of the kavalactones correlated poorly with their effects on AR protein down-regulation. C4-2B cells, an androgen-independent LNCaP derivative, were more sensitive to the AR down-regulating effect of the kavalactones and the kava extract than were LNCaP cells.

We next examined the stability of AR protein by treating the cells with cycloheximide to inhibit new protein synthesis. Figure 3D demonstrates that AR protein exhibits an accelerated degradation in C4-2B cells treated with either the kava extract or 5′6′-dehydrokawain, when compared with its stability in vehicle-treated cells. At 16 and 24 hours of treatments, the kava extract and 5′6′-dehydrokawain treatments decrease AR protein levels by about 80% and 70%, respectively, while the AR protein level in vehicle control only decrease by about 20% and 37%, respectively (Figure 3D).

### Flavokawain B down-regulates the expression of AR and its target genes (PSA and TMPRSS2)

**Figure 4:**
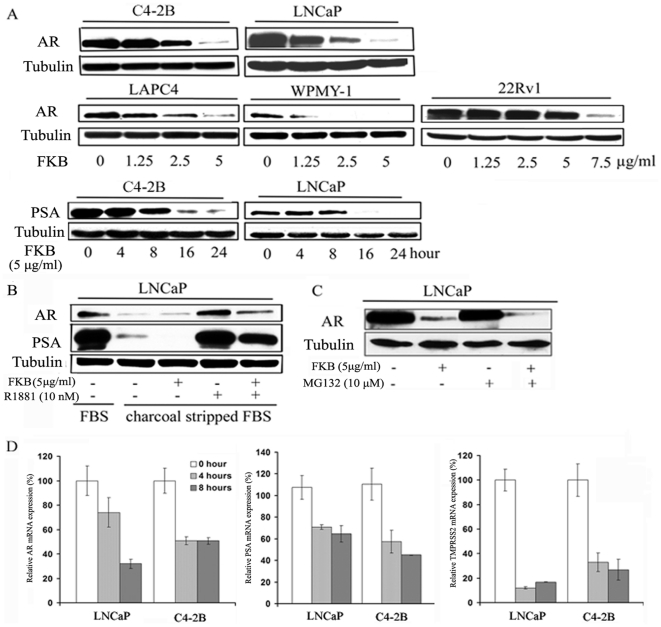
Flavokawain B (FKB) down-regulates mRNA and protein expression of AR and AR target genes. (**A**).The protein expression of AR and PSA after indicated treatments for 16 hours or 5 µg/ml FKB for indicated period of times was analyzed by Western blot. α-Tubulin was detected as a loading control. A representative blot was shown from three independent experiments. (**B**).Western blotting analysis shows that FKB decreases both constitutive and a synthetic androgen, R1881, stimulated AR and PSA expression in LNCaP cell. LNCaP cells were cultured in charcoal stripped FBS medium. FKB and R1881 at indicated concentrations treated cells for 16 hours. α -Tubulin was detected as a loading control. A representative blot was shown from three independent experiments. (**C**). Proteasome inhibitor, MG132, does not affect FKB mediated down-regulation of AR protein expression. LNCaP cells were treated with MG132 for 2 hours and then added FKB for additional 16 hours. α-Tubulin was detected as a loading control. A representative blot was shown from three independent experiments. (**D**). LNCaP and C4-2B cells were treated with 5 µg/ml FKB for 4 and 8 hours. Real-time RT-PCR was performed to analyze mRNA expression of AR and AR target genes (*PSA* and *TMPRSS2*). Bars are mean ± SD of three independent experiments. FKB significantly decreases mRNA expression of AR target genes (Student *t* test, P<0.01).

Flavokawain B is the most potent agent for inhibiting cell growth among the examined bioactive components of the kava extract. We therefore investigated the effect of flavokawain B on AR expression. Figure 4A shows that flavokawain B significantly decrease AR protein expression in all AR expressing cell lines (LAPC4, C4-2B, WPMY-1, LNCaP and 22Rv1). Interestingly, the AR protein level in WPMY-1 cells is the most sensitive to flavokawain B treatment. Since WPMY-1 represents a transformed prostate myofibroblast line [12], this result suggests that flavokawain B may be a novel agent for targeting PCa stromal AR. Flavokawain B also decreased the protein levels of PSA (Figure 4A). Figure 4B shows that flavokawain B inhibits the expression of AR and PSA proteins induced by a synthetic androgen analog, R1881. Pre-treatment with a proteasome inhibitor, MG132, did not recover the flavokawain B down-regulated-AR protein level (Figure 4C). In contrast to the kava extract and kavalactones treatments, treatment of LNCaP and C4-2B cells with 5 µg/ml flavokawain B for 4 and 8 hours resulted in a marked decrease in the mRNA levels of *AR* and its target genes *(PSA and TMPRSS2)* (Figure 4D). These results suggest that flavokawain B-mediated AR down-regulation is through its transcriptional regulation.

### The transcriptional suppression of AR by flavokawain B requires the expression of transcriptional factor Sp1

**Figure 5:**
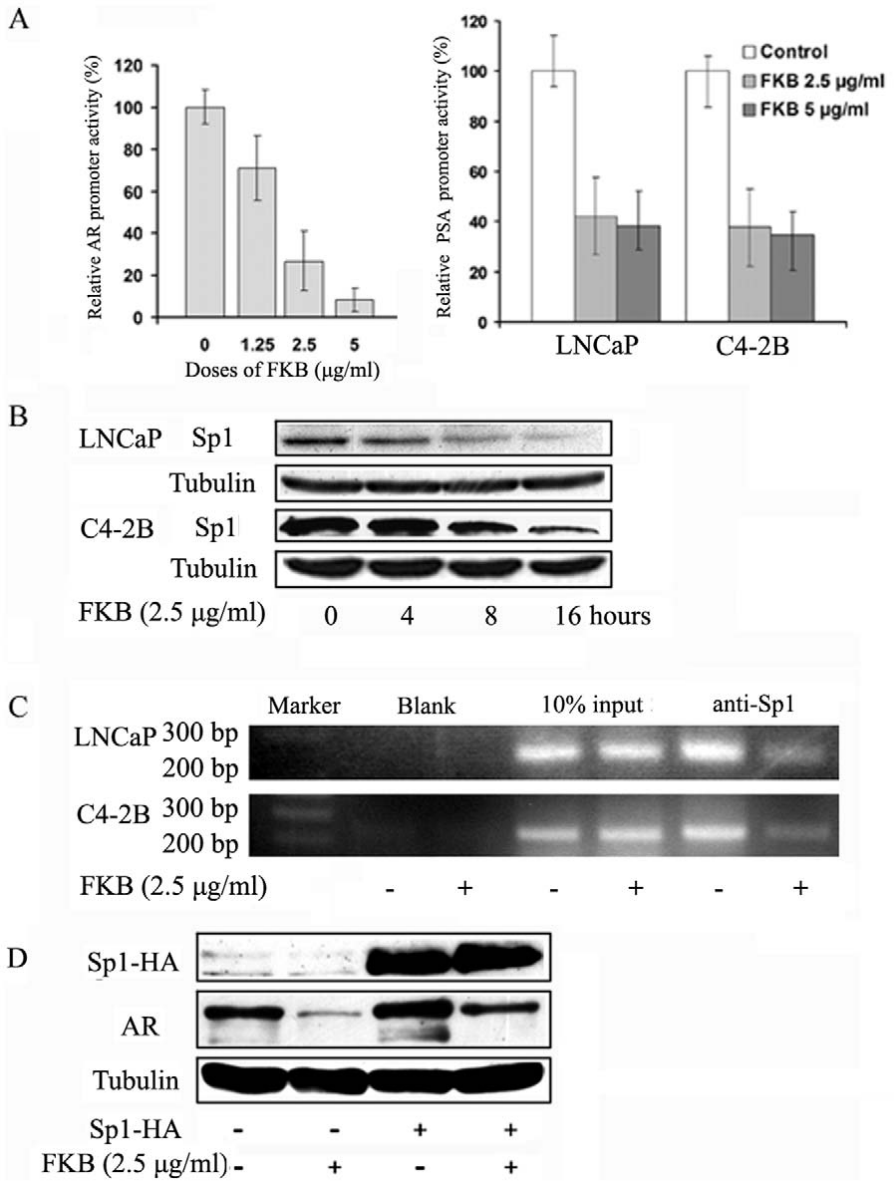
FKB mediated-AR transcriptional repression requires the expression of Sp1. (**A**).C4-2B cells were co-transfected with PSA-Luc or plARS-Luc, along with a Renilla luciferase plasmid pGL 4.71 and Luciferase activities were measured. FKB significantly decreases promoter activities of *AR* and *PSA* genes (Student *t* test, P<0.01). Bars are mean ± SD of three independent experiments. (**B**).Western blotting analysis of Sp1 protein expression. A representative blot was shown from three independent experiments. α -Tubulin was detected as a loading control. (**C**). ChIP analysis of the binding of Sp1 to the AR promoter sequence reveals that 16 hours FKB treatment results in a significant decrease in the binding of Sp1 to the AR promoter sequence that contain two Sp1 binding sites. Mouse IgG was used as a blank control. (**D**). Overexpression of Sp1 in C4-2B cells attenuates FKB induced AR protein down-regulation. After 48 hours of Sp1-HA plasmid transfection, C4-2B cells were treated with FKB for 16 hours. Sp1 and AR protein levels were examined. A representative blot was shown from three independent experiments. α -Tubulin was detected as a loading control.

We next investigated potential mechanism of flavokawain B mediated AR transcription. Sp1 is a putative transcriptional factor for AR regulation. Sp1 directly binds to the GC-box in the AR promoter through a zinc-finger protein motif [13], [14]. Therefore, an AR promoter luciferase reporter that contains four Sp1 binding sites was used to assay AR promoter activity in PCa cell lines. Figure 5A shows that flavokawain B treatments markedly decrease AR promoter activity in a dose-dependent fashion. The decrease in AR promoter activity by flavokawain B was associated with a reduction of Sp1 protein levels (Figure 5B). Consequently, ChIP analysis revealed that flavokawain B treatment of C4-2B and LNCaP cells markedly inhibited the binding of Sp1 to the AR promoter sequences (Figure 5C). To examine whether Sp1 is required for flavokawain B-mediated AR down-regulation, C4-2B cells were transiently transfected with Sp1 and then treated with flavokawain B. Figure 5D shows that overexpression of Sp1 increased AR protein levels and attenuated the flavokawain B-induced AR down-regulation. Taken together, our results clearly demonstrate that flavokawain B decreases Sp1 protein expression, leading to AR transcriptional down-regulation.

### Kavalactones and flavokawain B combination results in an enhanced inhibitory effect on growth of C4-2B cells and on expression of AR protein

**Figure 6:**
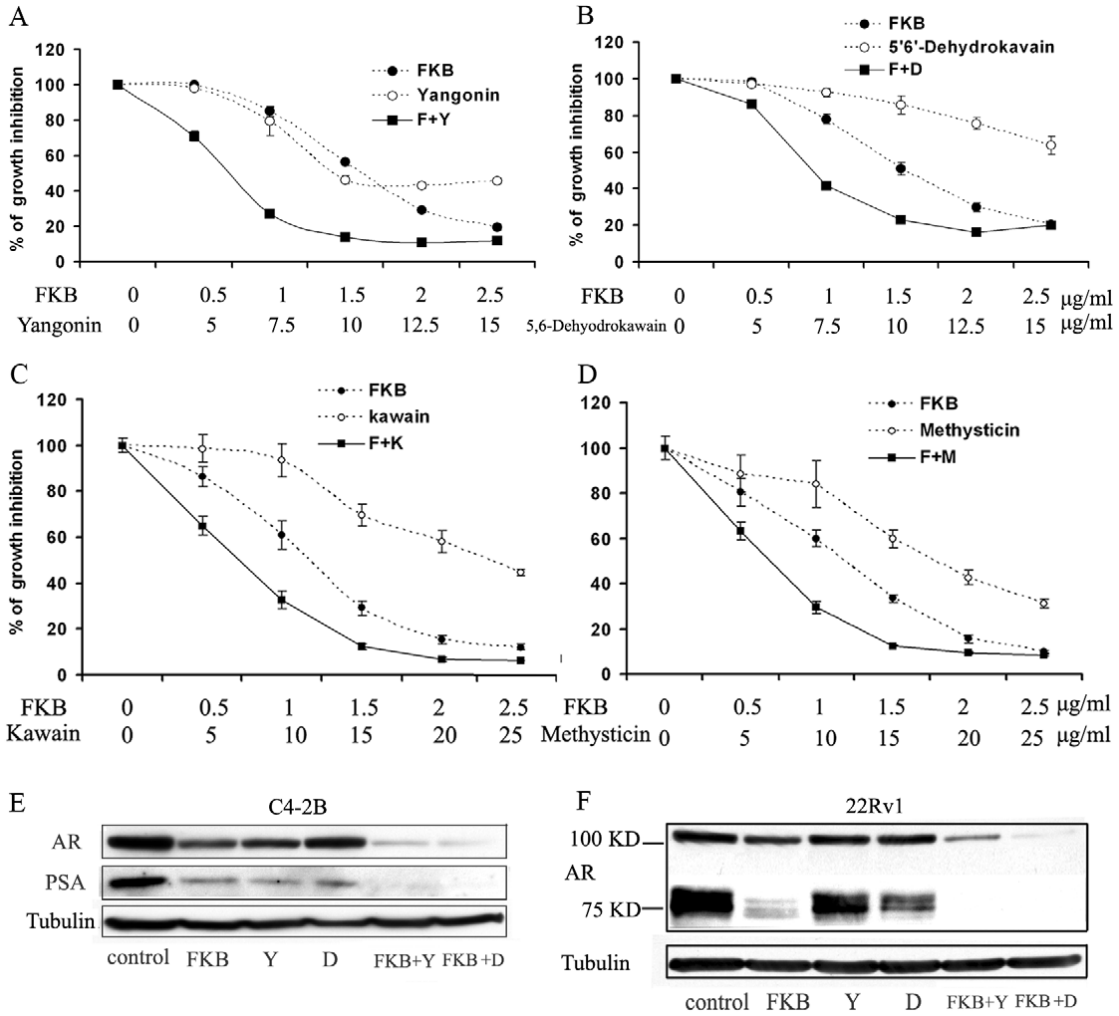
FKB and yangonin or 5′6;-dehydrokawain act synergistically to reduce the growth of C4-2B cells and down-regulate the expression of the full-length AR and an AR splice variant. The growth inhibitory of FKB and yagonin (**A**), or 5′6;-dehydrokawain (**B**), or kawain (**C**), or methysticin (**D**) alone and in combination on C4-2B cells. Each point is the mean of four independent experiments; bars, SD. Each sample was counted in duplicate. IC_50 s_ were estimated by dose-response curves, IC_50 s_ of the combinations are significantly lower than those of the treatments alone (Student *t* test, P<0.01). (**E**) **and** (**F**) Western blotting analysis of expression of the full-length AR (110 KDa), the AR splice variant (83 KDa) and PSA in C4-2B and 22Rv1 cells, respectively. A representative blot was shown from three independent experiments. α -Tubulin was detected as a loading control.

Figure 6A and B shows that 7.5 µg/ml yangonin and 1 µg/ml flavokawain B alone inhibited the growth of C4-2B cells by 15% and 20%, respectively, while their combination reduced the growth by more than 70%. Similarly, 5′6′-dehydrokawain at a dose of 7.5 µg/ml decreased the growth of C4-2B cells by less than 5%, while its combination with 1 µg/ml flavokwain B inhibited the growth by about 60%. Consistent with this result, flavokawain B in combination with other kavalactones (i.e. kawain and methysticin) also resulted in a synergistic inhibitory effect on the growth of C4-2B cells (Figure 6C and D). Furthermore, combination of 5′6′-dehydrokawain or yagonin with flavokawain B treatments resulted in an enhanced down-regulation of AR protein expression in both C4-2B and 22Rv1 cells (Figure 6 E and F). Notably, 22Rv1 harbors an AR splicing variant with a molecular weight of about 80 KD [15]. Flavokawain B and 5′6′-dehydrokawain are more effective in decreasing the expression of the AR splicing variant than that of the AR full length (Figure 6F). These results indicate that kavalactones and flavokawain B act synergistically or additively to inhibit growth of PCa cells and to down-regulate protein expression of AR and the AR splice variant.

### Kava extract and flavokawain B decrease the growth of patient-derived PCa xenografts in SCID mice, AR expression in tumor tissues and serum PSA levels

To determine the *in vivo* anti-PCa effect of the kava extract and flavokawain B, we developed two more clinically relevant, patient-derived PCa xenograft models, GM0308 and RC0309. The GM0308 patient-derived PCa xengrafts line was derived from a high-grade (gleason score sum = 5+5) PCa prostatectomy specimen obtained from a patient who was previously treated with androgen deprivation therapy (ADT), docetaxel plus carboplatin, and etoposide plus cisplatin. The xenograft tumor growth was initially established by using a subrenal xenograft technique, and then passed through subcutaneous injection of tumor pieces. The RC0309 line was derived from a high-grade (gleason score sum = 5+4) PCa prostatectomy specimen obtained from a patient who had no previous treatment. These human cancer xenografts faithfully preserve the histopathological characteristics of the original clinical sample. Both lines secrete PSA into the host mouse serum (Figure 6A). The GM0308 line expresses a truncated AR around 80 KDa, without missense mutations in exons 3, 4, or 5 (data not shown). In contrast, RC0309 holds the full length AR (data not shown).

**Figure 7:**
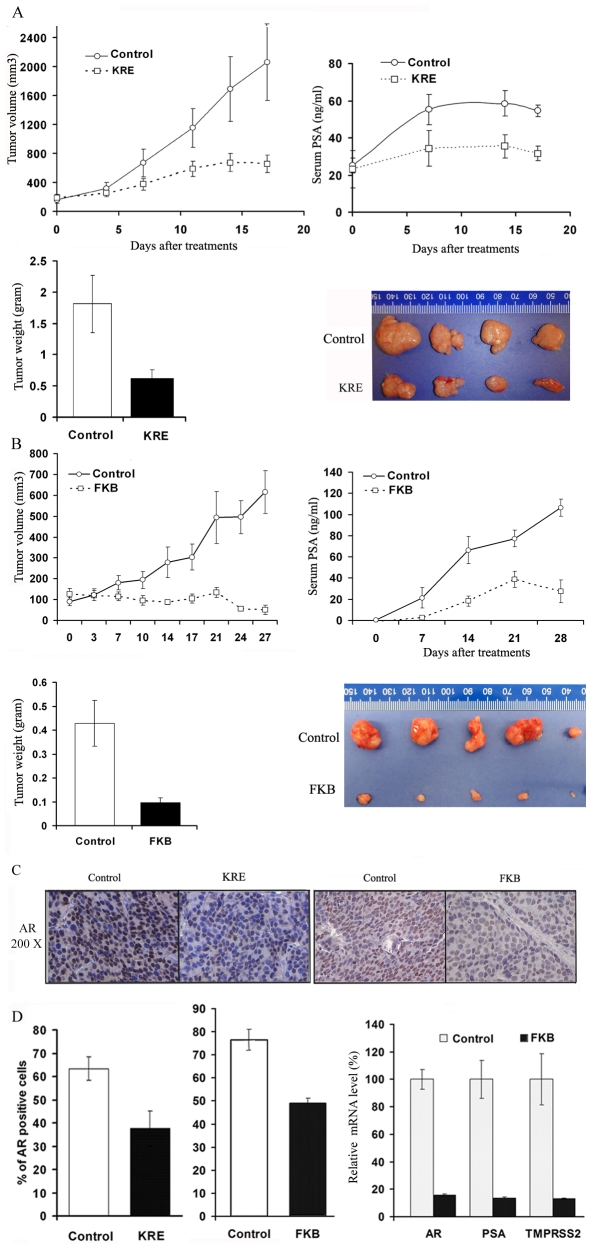
Kava root extract and FKB inhibit tumor growth, reduce serum PSA levels and decrease AR expression in tumor tissues in patient-derived PCa xenograft models. (**A**) and (**B**). The mice bearing patient-derived PCa tumors were randomly divided into treatment and control groups with similar pre-treatment serum PSA levels in each group. Tumor volumes and serum PSA were recorded, and presented as mean ± SD. Wet weight of tumors is represented as mean of tumors from individual mouse in each group. Bars, means ± SD. The tumor volumes (repeated-measures analysis of variance (ANOVA), p<0.01) and tumor weights (Student *t* test, P<0.01) of FKB and the KRE treated mice were significantly lower than those of vehicle control treated mice. (**C**). Immunohistology staining of AR expression in tumor tissues. Control immunostaining was performed with IgG isotype alone; Slides were counterstained with hematoxylin and photographed using a light microscope. Original magnification: ×200. (**D**). AR positive cells were counted in 12 fields in each group. The percentage of AR positive cells was calculated and presented as mean ± SD (the left two panels). The mRNA levels of *AR*, *PSA* and *TMPRSS2* in tumor tissues treated with vehicle or FKB were analyzed by RT real-time PCR (the right panel). Bars, means ± SD. The percentages of AR positive cells are significantly lower in the KRE and FKB treated groups than those in vehicle control treated groups (Student *t* test, P<0.01). The mRNA levels of *AR*, *PSA* and *TMPRSS2* is lower in the KRE and FKB treated groups than those in control groups (Student *t* test, P<0.01).

Mice bearing GM0308 tumors at passage 3 were treated with vehicle control or 200 mg/kg flavokawain B daily by IP injection for 28 days. Figure 6A shows a progressive tumor xenograft growth and serum PSA rise during the entire study in vehicle control group. The flavokawain B treatment, however, resulted in a decreased rate of tumor growth compared with control group throughout the study (Figure 7A). The wet tumor weights or serum PSA in control and flavokawain B–treated group recorded at the end of the treatment are 0.43±0.27 g and 0.097±0.048 g, or 66.6±12.8 ng/mL and 18.4±4.8 ng/ml, respectively (mean ± SD; n = 13 for control and n = 6 for FKB treatment groups; P<0.001, Student's t test). Flavokawain B treatment attenuated tumor growth by 77.3% and decreased the serum PSA levels by 68% at the end of the treatment.

Mice bearing RC0309 tumors at passage 13 were fed with food supplemented with vehicle control or 6 g/kg the kava extract for 18 days. Similarly, the kava root extract supplementation resulted in a significant decrease in the growth rate of tumors compared to vehicle control (Figure 7B, ANOVA, P<0.01). The wet tumor weights were 1.83±0.79 g in the control group and 0.73±0.34 g in the kava root extract group (Figure 7B; n = 5, mean ± SD; P<0.05, Student's t test). The kava extract attenuated tumor growth by approximately 60.1% and decreased the serum PSA levels by 42% at the end of the treatment.

Immunohistochemical analysis shows that tumor sections from the kava extract or flavokawain B treated PCa xenografts exhibited a significant decrease in both density and the number of positive AR staining cells compared to those of vehicle control treatments (Figure 7C). Consistently with *in vitro* results (Figure 4C), flavokawain B reduced the mRNA expression of AR and its target genes (*PSA* and *TMPRSS2*) by about 84%, 86% and 87%, respectively, compared to vehicle control treatments (Figure 7D).

**Figure 8:**
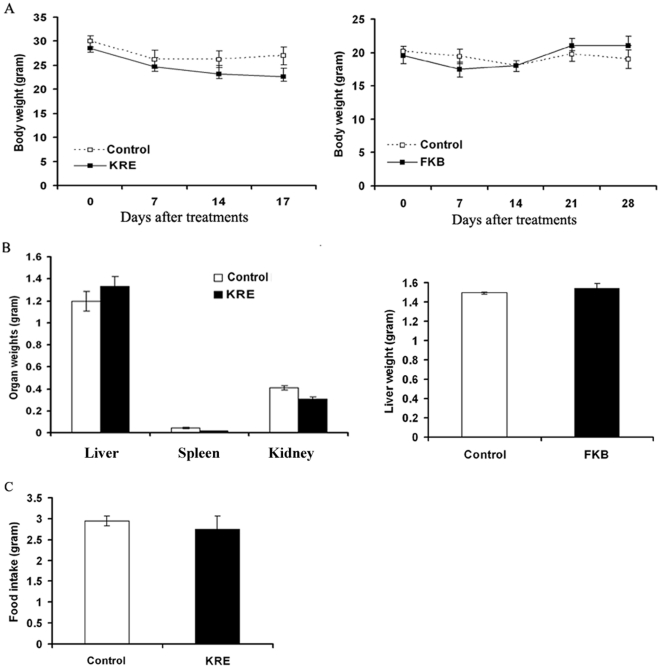
The effect of the kava root extract and FKB on mouse body and organ weight and food intake. (A). Body weights of vehicle, the kava root extract, and FKB treated SCID mice were recorded weekly and presented as mean ± SD. (B). Organ weights of vehicle, the kava root extract, and FKB treated SCID mice at the end of the experiments were recoded and presented as mean ± SD. (C). Food intakes of vehicle and the kava root extract treated SCID mice were recorded weekly and presented as mean ± SD.

The body weight gain and diet and water consumption of the flavokawain B–treated mice were similar to the control group of mice (Figure 8, right panels, Student t test, Ps>0.05). In addition, the mice did not show any gross abnormalities on necropsy. However, dietary feeding of the commercial kava extract resulted in a decrease in food up-take, wet spleen and kidney weight, and body weight, as well as an increase in wet liver weight (Figure 8, left panels; Student t test, Ps<0.05). These results suggest that unidentified components of the kava extract, but not flavokawain B, may be toxic to immunodeficient mice.

## Discussion

Androgens and the AR play an essential role in both early and late PCa. Current approaches targeting androgens/AR in PCa include treatment with gonadotropin receptor agonists and antagonists [16], inhibition of 5α-reductase [17], [18], inhibition of the steroidogenic enzyme CYP17A1 [19], blockade of AR ligand binding (e.g. MDV3100) [20]–[22] and the AR N-terminal domain binding (e.g. EPI-001) [23]. Although most of these approaches have demonstrated their effectiveness in prevention and/or treatment of PCa and Castration-resistant prostate cancer (CRPC) in clinics, there are several situations that lead to resistance to the treatments and worsening disease. First, PCa grows in a complex and heterogeneous environment, which include different types of cells (e.g. androgen-dependent and independent PCa cells and stromal cells). Targeting only androgen-dependent PCa cells would allow over-population of other types of cells that are not responsive to the androgen/AR targeting approaches described above. Second, ADT and the inhibitor of CYP17A1 have been demonstrated to cause AR overexpression and generation of AR splice variants that lack the ligand binding C-terminal domain [19], [24]. These treatments would confer ligand-independent AR transactivation. Suppression of AR expression therefore would become the straight-forward but efficient strategy for PCa therapy. Third, androgen-driven stromal-epithelial interactions play an important role in PCa [25]. Targeting stromal AR would have potentials for PCa prevention and treatment. Therefore, agents that can potently inhibit the growth of both androgen-dependent and -independent PCa cells and suppress the expression of AR splice variants and stromal AR are highly desirable, novel and of clinical translational values.

We demonstrate that kava extract, kavalactones and flavokawain B can down-regulate the expression of both the full-length AR and AR splice variants with more potency to expression of an AR splice variant. In addition, flavokawain B decreases AR expression in a transformed prostate stromal cell line WPMY-1. The growth inhibitory effect of the kava extract, kavalactones and flavokawain B appears not to correlate to AR expression in PCa cell lines. It is possible that the kava extract, kavalactones and flavokawain B also have other targets for their growth inhibitory effect. Taken together, these results suggest that the kava extract and its active components, specifically flavokawain B, deserve further investigation of their usefulness in treatment of PCa and CRPC in particular.

The patient-derived PCa xenografts are thought to more accurately preserve the histopathological and genotypical characteristics of the original clinical samples [11], [26] than do cell line-derived xenografts. Therefore, the use of this model system in our study would be more clinically relevant than the current use of cell lines and transgenic mouse model of PCa. We demonstrate here that both the kava extract and flavokawain B markedly reduce tumor weight by about 60 and 77% and serum PSA levels by about 42% and 68%, respectively. The activity of flavokawain B against GM0308 tumors is especially noteworthy, since this tumor was derived from a patient who was previously treated with ADT, docetaxel plus carboplatin, and etoposide plus cisplatin. These data suggest that flavokawain B may act through novel target molecules to kill cancer cells of patients with advanced PCa resistant to conventional therapy [27].

The mechanisms of kavalactones and flavokawain B mediated AR down-regulation are different. Kavalactones act through a mechanism of AR protein degradation, while flavokawain B down-regulate Sp1 expression leading to a decrease of *AR* mRNA levels. In addition, the kavalactone and flavokawain B combination results in an enhanced inhibitory effect on PCa cell growth and AR expression. The transcriptional regulation of *AR* gene remains largely unknown. Several groups reported that Sp1 is a key regulator of *AR* gene transcription [13], [14]. We demonstrated that flavokawain B decreased Sp1 expression and its binding to the *AR* promoter. However, it is still unclear why flavokawain B is more potent to androgen-independent cell lines. A recent study showed that purine-rich element binding protein (PUR) alpha binds to a site near Sp1 in the AR promoter and suppress AR expression, and that PUR alpha expression was associated AR overexpression in androgen-independent cell lines [28]. Therefore, the further investigation on the interaction between Sp1 and PUR alpha under flavokawain B treatment may provide a novel mechanism for flavokawain B induced AR down-regulation. In addition, the mechanism of kavalactones induced AR protein degradation would also deserve further investigation.

In summary, the kava root extract and flavokawain B inhibited tumor growth in clinically relevant xenograft models, decreased AR expression in tumor tissues, and lowered serum PSA levels in tumor-bearing mice. The down-regulation of AR expression by kavalactones is associated with protein degradation. In contrast flavokawain B decreases expression of both the full-length AR and AR splice variants via inhibition of Sp1 mediated AR transcription. Flavokawain B is more potent to castration-resistant PCa. Since traditional kava preparation has been safely consumed for thousands of years, it is unlikely that active components of kava extracts, including kavalactones and flavokawains, are all toxic to human. Therefore, the results obtained from this study clearly suggest a need for further developing a safe and inexpensive kava product or its active components for treatment and prevention of PCa and CRPC.
